# The CYPome of the model xenobiotic-biotransforming fungus *Cunninghamella elegans*

**DOI:** 10.1038/s41598-019-45706-x

**Published:** 2019-06-25

**Authors:** William Palmer-Brown, Raúl Miranda-CasoLuengo, Kenneth H. Wolfe, Kevin P. Byrne, Cormac D. Murphy

**Affiliations:** 1 UCD School of Biomolecular and Biomedical Science, University College Dublin, Belfield Dublin, 4 Ireland; 2 UCD School of Medicine, Conway Institute, University College Dublin, Belfield Dublin, 4 Ireland

**Keywords:** Biotechnology, Microbiology

## Abstract

The fungus *Cunninghamella elegans* is recognised as a microbial model of mammalian drug metabolism owing to its ability to catabolise xenobiotic compounds in an analogous fashion to animals. Its ability to produce phase I (oxidative) metabolites of drugs is associated with cytochrome P450 (CYP) activity; however, almost nothing is known about these enzymes in the fungus. In this paper we report the *in silico* analysis of the genome sequence of *C*. *elegans* B9769, which contains 32 genes putatively coding for CYPs. Based on their predicted amino acid sequences these were classified as belonging to CYP509, 5203, 5208, 5313, 5210, 61 and 51 families. Reverse transcription-quantitative PCR revealed that the gene coding for CYP5313D1 was significantly upregulated when *C*. *elegans* DSM1908 was cultivated in sabouraud dextrose in contrast to its expression in cells grown in Roswell Park Memorial Institute medium. This corresponded to the fungus’ xenobiotic biotransformation ability when grown in the two media. Heterologous expression of *cyp5313D1* in *Pichia pastoris* resulted in a recombinant strain that biotransformed flurbiprofen to 4′-hydroxyflurbiprofen, the same metabolite generated by *C*. *elegans* cultures. This is the first report of a xenobiotic-biotransforming CYP from this biotechnologically important fungus.

## Introduction

*Cunninghamella* spp. can biotransform drugs and other xenobiotics to generate both oxidative (phase I) and conjugative (phase II) products that are similar to those identified in humans and other mammals^1^. Thus fungi in this genus, in particular *C*. *elegans*, *C*. *blakesleeana* and *C*. *echinulata*, have been extensively studied as models of mammalian xenobiotic metabolism^2^. Oxidative/phase I metabolites of drugs and xenobiotics are formed via the actions of cytochromes P450 (CYPs); in humans there is a small subset of the 57 CYP isoforms (principally composed of CYP1A2, 3A4, 2C9, 2D6 and 2E1) that is responsible for catalysing the oxidation of xenobiotics^3^. Some bacterial CYPs are also known to biotransform xenobiotics into phase I metabolites, for example, *Actinoplanes* sp. ATCC 53771 can convert diclofenac to the mammalian metabolite 4′-hydroxydiclofenac^4^. Xenobiotic-transforming CYPs in other fungi have been studied^5,6^ and several fungal CYPomes are characterised^7–9^, but the CYPome of *Cunninghamella* spp. is largely unexplored.

The presence of CYPs in *Cunninghamella* spp. has been inferred from the oxidative metabolites formed in the presence of different drugs and from inhibitor studies. For example, Zhang *et al*.^10^ investigated the biotransformation of amitriptyline in *C*. *elegans* and found that in the presence of known P450 inhibitors such as 1-aminobenzotriazole, metabolism of the drug was reduced by 95%. However, direct evidence of CYP activity in the fungi is scarce with limited reports of *in vitro* CYP-catalysed biotransformation of xenobiotics in cell-free extracts^11^. One *Cunninghamella* CYP gene has been cloned and overexpressed in *E*. *coli* and the protein confirmed to be a CYP by immunological methods^12^. However, no biochemical assays were conducted and its sequence places it in the family CYP509, members of which are not known to be involved in xenobiotic biotransformation^13^. A cytochrome P450 reductase (CPR) has also been identified in *C*. *elegans* and its expression demonstrated by northern blot^14^. Expression of the *cpr* and *cyp509A1* has been measured under different culturing conditions by other researchers^15–17^ and these studies have shown their up-regulation in the presence of xenobiotic compounds. However, the absence of a defined function of the proteins is unsatisfactory, in particular given the central role of CYPs in phase-I metabolism and their value as biocatalysts.

Recently, the unannotated genome sequence of *Cunninghamella elegans* B9769 was published, providing access to sequence data for this important microorganism. In this paper we report the *in silico* analysis of the genome to identify the CYPome of the fungus, the expression of different CYPs under conditions known to influence xenobiotic biotransformation and the identification of a xenobiotic-transforming CYP by heterologous expression.

## Results

### Prediction of the CYPome of *C*. *elegans*

The genome of *Cunninghamella elegans* B9769 (Accession Number: JNDR01001308.1) was sequenced as part of a comparative *Mucor* spp. genome analysis project^18^. The length of the genome is 31,743,477 bp. The generalised hidden Markov model (GHMM) *ab initio* program AUGUSTUS (http://augustus.gobics.de/) was used to produce a probabilistic model of the genome sequence and its gene structure^19^. The gene-finding parameters were established using *Schizosaccharomyces pombe* as a species-specific training set. The predicted genes were then used to create a database to query for nucleotide (BLASTN) and protein (BLASTP) analysis. A total of 8028 protein coding genes were predicted.

The predicted amino acid sequences were examined using published sequences from UniProt of known xenobiotic CYPs for BLASTP analysis^20^. Sequence similarity searches were performed using mammalian CYPs and the microbial CYPs 53A1 (*Aspergillus niger*), 53A19 (*Fusarium oxysporum*) and 509A1 (*Cunninghamella elegans*). A total of 53 sequences were detected in the predicted proteome of *C*. *elegans* B9769 as homologous hypothetical proteins based on their sequence similarity to the reference CYPs. For example, the predicted homolog of the previously identified CYP509A1 shared a sequence identity of 98%. However, a second search focusing on the presence of conserved sequence motifs reduced the number of CYP candidates to 32, which were formally classified (Table 1). Most fell into known fungal CYP families (51, 509, 5206, 5313, 5203, 61, 5210 and 5205); one new family was identified (CYP5876) and one new sub-family (CYP509M1). The remaining 21 sequences were either partial proteins, inferred by their shorter length (<300 amino acids) or were identified as other enzymes when queried using BLASTP on GenBank®.

**Table 1:** The CYPome of *Cunninghamella elegans*.

Gene no.	CYP name	Length (aa)	Haem-binding domain		PERF domain		K-helix			
			Start (aa)	FXXGXRXCXG	Start (aa)	PXRX	Start (aa)	EXXR	CYP ortholog (% identity)	Organism
5706	5208A3	523	450	FSTGRRVCVG	421	PERW	366	ETLR	5208A2 (54)	*Mucor*
7634	5876A1	451	363	FGFGPRHCPG	337	PERF	282	ECLR	5206A9 (32)	*Mucor*
5134	5206V1	400	332	FGWGRRICPG	306	PNRF	263	EVLR	5206J2 (41)	*P*. *blakesleeanus*
5707	5206W1	349	298	FGWGRRICPG	272	PDRF	217	ECLR	5206A3 (39)	*Rhizopus oryzae*
5705	5206U3	393	347	FGWGRRICPG	321	PDRF	266	ESLR	5206D1 (43)	*P*. *blakesleeanus*
6050	5206U2	428	353	FGWGRRICPG	327	PDRF	281	ESLR	5206A9 (40)	*Mucor*
6049	5206U1	501	440	FGWGRRICPG	414	PDRF	264	EALR	5206Q1 (44)	*Mucor*
3940	5313E1	511	447	FSLGSRNCIG	427	PERW	371	ETFR	5313A5 (47)	*Mucor*
2620	5313D1	512	446	FSAGSRNCIG	427	PERW	371	ETMR	5313A5 (47)	*Mucor*
5881	61A1	496	453	FGSGPRHCIG	434	PDRW	379	EVLR	61A1 (80)	*Rhizopus oryzae*
2271	51	512	451	FGAGRRHCIG	412	PYRW	355	ETLR	51 (75)	*P*. *blakesleeanus*
7636	5203A23	498	446	FHAGPRVCLG	425	PERW	375	ESLR	5203 (60)	*Mucor*
4152	5203A19	516	455	FHGGPRVCLG	434	PERW	378	EVLR	5203 (61)	*Mucor*
4151	5203A18	516	455	FHGGPRVCLG	434	PERW	378	EVLR	5203 (60)	*P*. *blakesleeanus*
4150	5203A17	515	454	FHGGPRFCLG	433	PERW	377	EVLR	5203A13 (61)	*R*. *oryzae*
4156	5203A20	509	448	FHGGPRVCLG	427	PERW	371	EVLR	5203A14 (65)	*Mucor*
2735	5203A16	381	318	FHAGPRVCLG	297	PERW	241	ETLR	5203A13 (62)	*Mucor*
6919	5203A22	506	446	FHAGPRVCLG	425	PERW	369	EVLR	5203A5 (57)	*R*.*oryzae*
4322	5203A21	526	466	FHAGPRVCLG	445	PERW	372	EVLR	5203A13 (59)	*Mucor*
4063	5210C1	580	519	FLYGPRTCIG	500	PDRW	445	ESLR	510A6 (51)	*P*. *blakesleeanus*
2194	5210B1	517	457	FLAGGRQCIG	438	PSRW	383	ESLR	510A6 (48)	*P*. *blakesleeanus*
4598	5205A8	1,211	482	FSTGPRACIG	463	PDRF	408	ETLR	5205A5 (62)	*Mucor*
**7756**	**509A1**	**474**	**409**	**FSSGNRQCIG**	**387**	**PDRW**	**331**	**ETLR**	**509A1 (94)**	***C***. ***elegans***
7465	509A4	497	460	FSNGSRQCLG	440	PDRF	384	ETMR	509A1 (60)	*C*. *elegans*
7464	509A5	398	362	FSNGSRQCLG	342	PDRF	286	ETMR	509A1 (61)	*C*. *elegans*
7761	509N1	549	449	FSSGPRMCIG	427	PDRF	371	ETLR	509A1 (48)	*C*. *elegans*
7762	509A3	345	281	FSNGSRQCIG	259	PDRF	219	ETLR	509A1 (60)	*C*. *elegans*
7258	509A2	302	304	FSKGNRQCIG	282	PERF	226	ETLR	509A1 (67)	*C*. *elegans*
6826	509Q1	538	474	FASGSRMCIG	452	PDRF	397	ETLR	509A1 (45)	*P*. *blakesleeanus*
2634	509P1	408	390	FSSGPRICIG	368	PDRF	313	ETLR	509F1 (45)	*P*. *blakesleeanus*
2635	509P2	585	308	FQHGPRVCIG	286	PDRF	231	ETLR	509F1 (50)	*P*. *blakesleeanus*
7342	509M1	453	398	FSSGQRQCVG	369	PERF	314	ENLR	509C9 (40)	*R*. *oryzae*

The table includes the conserved domains of the proteins and the closest homology to other fungal CYPs. The previously identified CYP509A1 is highlighted in bold.

Fungal CYPs are mainly class II^21^, which are membrane bound proteins and require a redox partner (see following section) to deliver electrons to the haem. Using the web-based algorithm SOSUI (http://bp.nuap.nagoya-u.ac.jp/sosui), the solubility of the *C*. *elegans* CYPs was investigated and curiously only five (5208A3, 5206U1, 51, 5203A22, 509Q1) were predicted to have a transmembrane region. It seems highly unlikely that so many of the CYPs would be soluble, since to date only one soluble fungal CYP, P450nor, has been identified^21^. Further biochemical analysis of the CYPs will help shed light on this anomaly.

Of particular interest for this study were the potential xenobiotic-biotransforming CYPs. Apart from CYP51 and CYP61, which are highly conserved in fungi and are involved in sterol biosynthesis^22^, the functions of the other CYPs are either unknown or not experimentally demonstrated. As *C*. *elegans* is known for mimicking mammalian phase I (oxidative) biotransformations, relatedness to mammalian CYPs was used as a key criterion to identify potential xenobiotic-biotransforming enzymes. CYPs 5313D1 and 5313E1 were of particular interest as their amino acid sequences showed comparatively high identity (approx. 31%) to the main mammalian CYP involved in drug detoxification, CYP3A4. In contrast, the previously cloned CYP509A1^12^ had a 24% identity to this enzyme. Furthermore, members of the CYP53 family in fungi are associated with 4-hydroxylation of benzoic acid and its derivatives^22^. In *C*. *elegans*, para-hydroxylation of aryl rings of xenobiotics such as diclofenac and flurbiprofen, is the predominant phase I pathway^23^. BLASTP searches of the predicted *C*. *elegans* proteome using the amino acid sequence of CYP53A1 from *A*. *niger* and CYP53A19 from *F*. *oxysporum* revealed that the highest similarity was to CYP5313E1 (26 and 31%, respectively).

### Identification of redox partner proteins

To identify cytochrome P450 reductases (CPRs), the known *C*. *elegans* CPR sequence^14,15^ was used as a sequence for BLASTP analysis. The hypothetical CPRs were checked for a flavin adenine dinucleotide (FAD)-binding domain and a NAD(P)H-dependent flavin mononucleotide (FMN) domain. Additionally, cytochrome b5 (Cyt-b5) and cytochrome b5 reductase (Cyt-b5r) sequences from *Phanerochaete chrysosporium*^24^ were used to identify possible alternative CYP redox partners.

Eight sequences were identified as possible NADPH reductase partners (Fig. S1). Three were identified as CPRs (Fig. S2), one of which (g4301) has been previously reported^14^. One sequence was identified as a NADPH-dependent diflavin oxidoreductase; however these enzymes are typically involved in the biogenesis of FeS proteins^25^. Four more sequences were identified as alternative redox partners and one sequence was recognised as a cytochrome b5. The remainder were identified as nicotinamide adenine dinucleotide (NADH) cytochrome b5 reductases.

### CYP expression in *C*. *elegans* DSM1908

**Figure 1:**
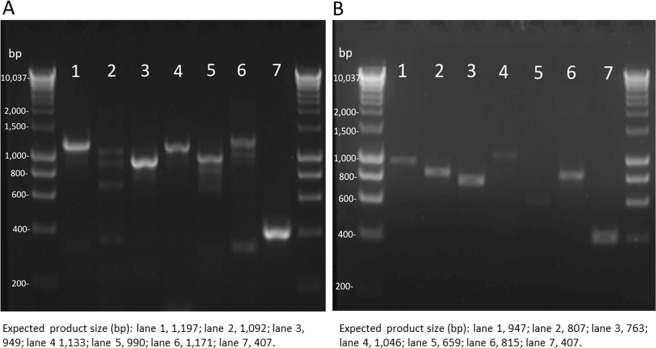
Amplification of CYP genes from *C*. *elegans* gDNA (**A**) and cDNA (**B**). The lanes are different CYPs whose primers are listed in Table S1: Lane 1, 5206U1; lane 2, 5208A2; lane 3, 5206D1; lane 4, 5205A8; lane 5, 509Q1; lane 6, 5210C1; lane 7, 5313E1. The expected amplicon sizes are given below the figure.

*C*. *elegans* DSM1908 is one of a number of strains that has been used by researchers to study drug metabolism^26,27^. To confirm that the genes identified in the in silico analysis of the *C*. *elegans* B9769 are also present in the DSM1908 strain, a set of internal primers were designed to amplify partial sequences of seven genes (Table S1); the genes chosen were selected based on their similarity to mammalian CYPs. These primers were used in PCR experiments with both genomic DNA and complementary DNA (cDNA) isolated from *C*. *elegans* DSM1980. PCR was performed initially using gDNA template and the targets were visualised by gel-electrophoresis, for which the expected amplicon sizes were detected (Fig. 1A). The same primers were then employed to investigate gene expression by amplifying targets from cDNA. As the internal primers were designed to span exon-exon boundaries, the products amplified from cDNA were smaller due to an absence of intron regions (Fig. 1B). Furthermore, only a single band was visualised by gel-electrophoresis representing an effective extraction of messenger RNA and subsequent reverse-transcription. The correct amplicon size also confirmed the accuracy of the gene prediction by supporting the position and length of the introns within the ORF. Finally, the amplicons were sequenced and confirmed that the genes were the same in both strains with >92% sequence identity. The sequence data were inputted into the genome browser Artemis and validated the correct position of the sequence.

Based on the visualisation of gene transcripts in Fig. 1B, CYPs 509Q1, 5206U1, 5206U3, 5205A8, 5208A3, 5201C1 and 5313E1 are expressed by *C*. *elegans* when cultivated for 72 h in the growth medium sabouraud dextrose broth (SDB), which is the medium often used to ensure good xenobiotic biotransformation in this fungus. Expression of CYP5313D1 under the same conditions was confirmed in a separate experiment (Fig. S3). Additionally, the cDNA amplicons reinforced the accuracy of the gene prediction through confirmation of correct product sizes.

### CYP5313D1 is up-regulated in *C*. *elegans* grown in sabouraud dextrose medium

A previous investigation demonstrated that when *C*. *elegans* biofilm was grown in Roswell Park Memorial Park (RPMI) medium, biotransformation of flurbiprofen was dramatically reduced compared with cells grown in SDB^28^. In the present study suspended cultures of *C*. *elegans* grown in these media showed the same pattern when incubated with the non-steroidal anti-inflammatory drug flurbiprofen (5 mg). Analysed by HPLC, 3.30 mg (66%) of 4′-hydroxyflurbiprofen was recovered from cultures grown in SDB after 72 h, whereas cells cultured in RPMI only yielded 0.1 mg (2%) of the metabolite.

This metabolite is the result of CYP monooxygenase activity, but to date the specific enzymes involved in the biotransformation are not known. The *in silico* analysis of the CYPome has revealed two CYPs (5313D1 and 5313E1) that may be involved in xenobiotic biotransformations. To investigate this further RT-qPCR was employed to look for changes in gene expression of *cyp5313D1* and *cyp5313E1* under the conditions optimal for biotransformation. In addition the change in expression of the previously identified *cyp509A1* and *cpr* was also measured. cDNA from four experimental conditions was prepared for investigation, which were *C*. *elegans* cultured in sabouraud dextrose broth and RPMI for 72 h and 96 h. Comparison of Cq values of technical replicates resulted in 86.9% falling within 0.5 Cq and 98.8% within 0.8 Cq. As expected, biological replication produced a higher level of variation with 50% of the replicates falling within 1.21 Cq and 95.8% within 2.65 Cq. Amplification efficiencies measured from serial dilutions of cDNA of all but the reference genes *cpr* and *sarA* were 1.87 or higher (Table S4). To normalise the sample data, a geNorm analysis was initiated on 11 samples and 3 reference targets (M = 0.440, V = 0.181). The genes *ubcB* (ubiquitin carrier protein) and *sarA* (secretion associated GTP-binding protein) were assessed as reference targets based on a previous study that identified a number of stable reference genes in filamentous fungi^29^. In addition, given its high expression stability, *cpr* was repurposed as a reference gene. The optimal number of reference targets in this experimental situation was 2 (geNorm V = 0.143): *ubcB* and *cpr*.

**Figure 2:**
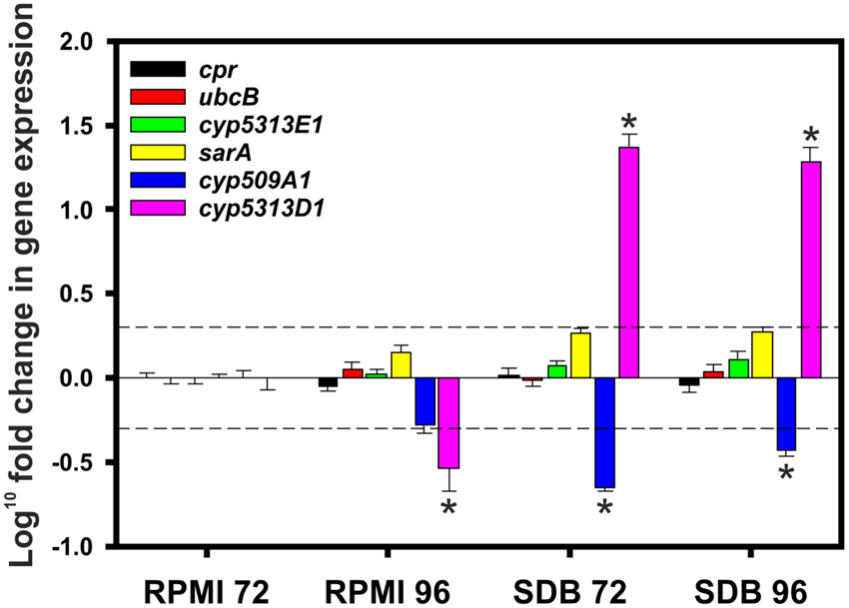
Relative quantification of CYP expression in *C*. *elegans* grown in media sabouraud dextrose or RPMI after 72 h and 96 h. Bars correspond to Log_10_ fold change in gene expression and error bars correspond to error propagation corrected standard errors. Broken lines show 2-fold changes to either side of the scale. Stars indicate significant changes in gene expression. The primers employed are given in Table S2.

The results shown in Fig. 2 were normalised to *ubcB* and *cpr* and calibrated against the RPMI 72 group, with positive and negative values showing up- and down-regulation of the gene of interest, respectively. The largest effect in the dataset was observed as a dramatic up-regulation of *cyp5313D1* in *C*. *elegans* when grown in SDB (~20 fold change, p < 0.0001). The same gene was 3.4 times down-regulated in the RPMI 96 group, possibly because the cells grown in this medium were under severe nutritional limitation at this time. The other genes remained consistently expressed in the various conditions (*cyp5313E1)* or were significantly down regulated in SDB (*cyp509A1*).

### CYP5313D1 is a xenobiotic-biotransforming enzyme

**Figure 3:**
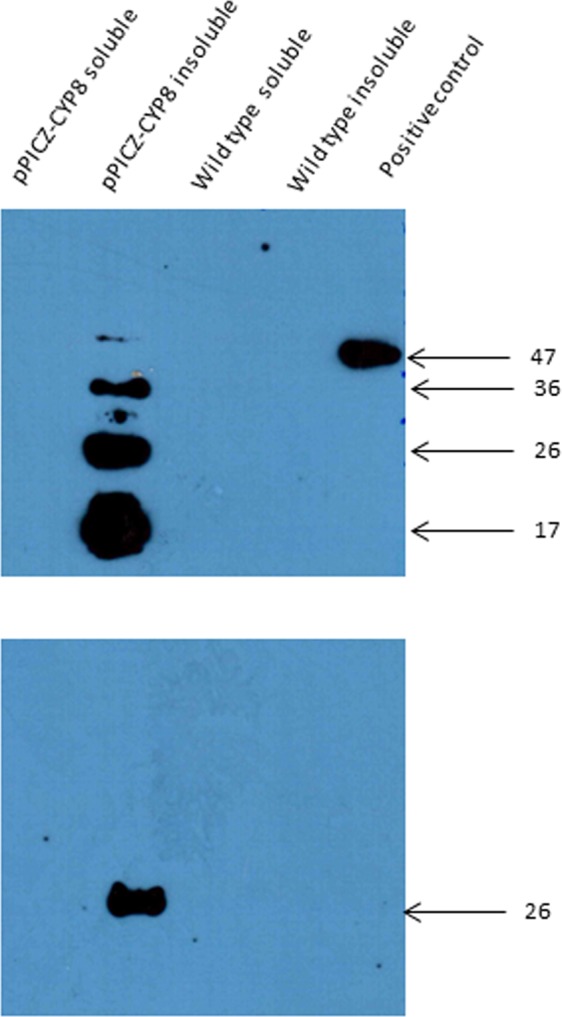
Expression of CYP5313D1 in *P*. *pastoris*. The proteins were detected with anti-His (upper blot) and anti-myc (lower blot) antibodies. Serpin B3 (his-tagged) was used as a positive control. The image was cropped for clarity; the full blot is shown in the Supplemental Information (Fig. S4).

The *in silico* analyses and expression studies suggested that CYP5313D1 is involved in phase I biotransformation of xenobiotics, thus the gene was overexpressed in a heterologous host. The yeast *P*. *pastoris* was selected as the host, since it has been used successfully in other studies with xenobiotic-biotransforming CYPs^30^ and has endogenous reductases. The gene coding for CYP5313D1 was cloned into pPICZ A without the N-terminal signal peptide so that the protein would not be exported from the cell, resulting in the expression plasmid pPICZ-cyp8. Positive transformants were confirmed by PCR. The recombinant strain was cultivated in BMY medium and protein expression was induced by the addition of methanol. Expression was confirmed by Western blot analysis of the lysate (Fig. 3) and showed that the expressed protein was localised in the insoluble fraction, which is consistent with fungal CYPs. Whilst proteins with both His- and myc- epitopes were detected in the recombinant *P*. *pastoris* cell extracts, only a faint band at the correct molecular weight (approx. 59 kDa) was detected using anti-his antibodies, indicating that extensive degradation of the protein had occurred.

**Figure 4:**
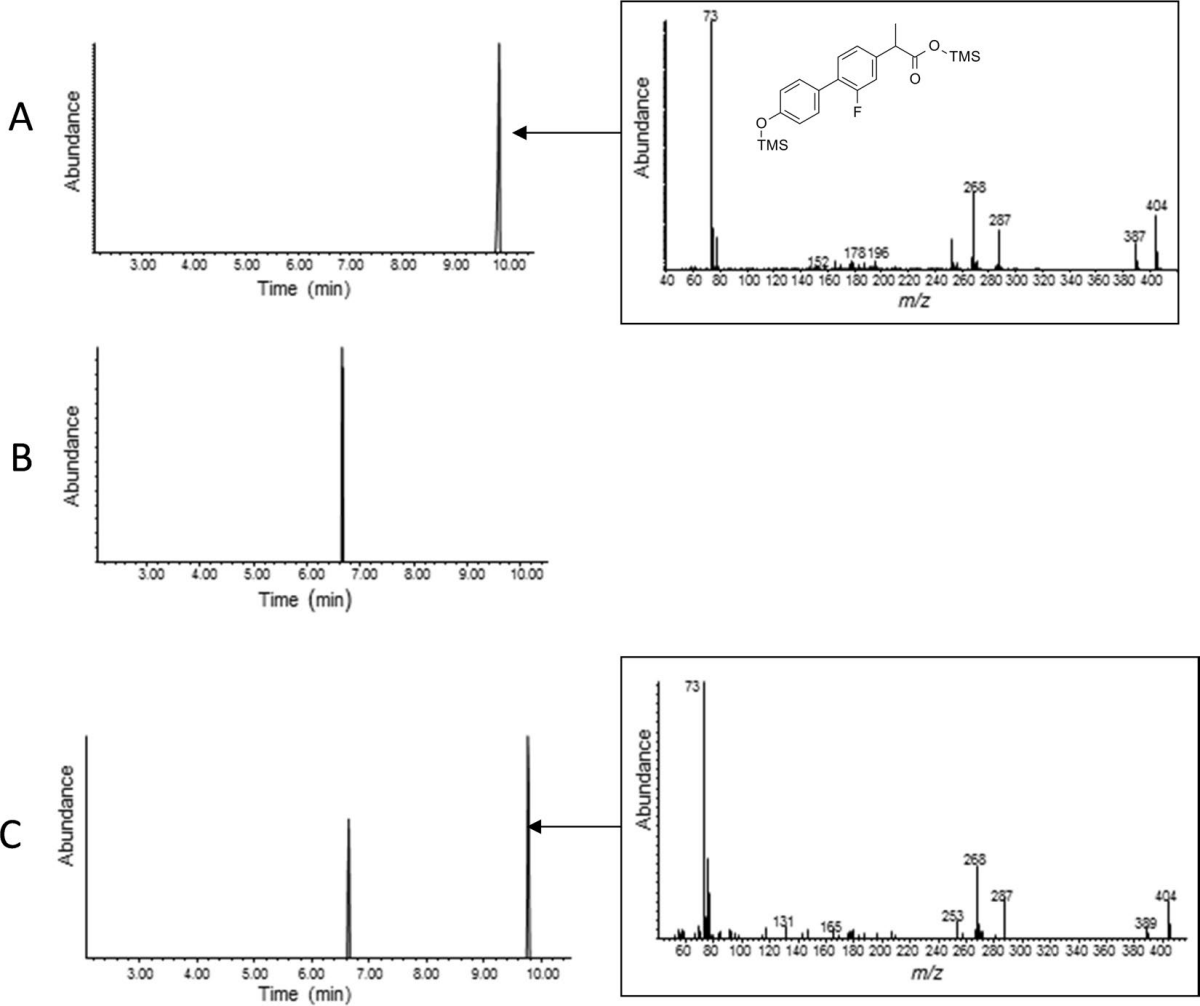
Biotransformation of flurbiprofen with *P*. *pastoris*. Selected ion chromatograms are shown (ion *m/z* 404, molecular ion of 4′-hydroxyflurbiprofen) for 4′-hydroxyflurbiprofen standard (**A**) *P*. *pastoris* wild type (**B**) and recombinant *P*. *pastoris* pPICZ-cyp8 (**C**).

Activity of recombinant CYP5315D1 was determined by adding the drug flurbiprofen to cultures after induction. The entire culture was extracted with ethyl acetate after 48 h incubation, and the extract dried and the residue silylated for GC-MS analysis. Figure 4 shows that after incubation with the drug, the metabolite 4′-hydroxyflurbiprofen was detected in extracts from the recombinant culture, whereas no 4′-hydroxylated metabolite was observed in control (wild type) cultures incubated with drug (Fig. 4B). The same metabolite was observed as the most prominent metabolite when *C*. *elegans* was incubated with flurbiprofen^26^, thus CYP5313D1 is the first xenobiotic-biotransforming CYP to be identified from the fungus.

## Discussion

*Cunninghamella* spp. have been studied for decades for their ability to produce phase I metabolites of drugs and other xenobiotics that are analogous to those produced in mammals^1^. Despite this, their CYPs have until now proven inaccessible. In this paper we identified the CYPome of the fungus *C*. *elegans* B9769 through *in silico* analysis of the genome, and found that there are 32 putative CYPs based on sequence homology and the presence of conserved motifs. The CYPs were classified according to the established methods. Two CYPs (5313D1 and 5313E1) were identified as putative xenobiotic-biotransforming enzymes, based on their sequence similarity to mammalian CYP3A4, which is involved in phase I metabolism, and members of the CYP53 family, which are known to catalyse hydroxylation of benzoic acid in basidiomycetes^31^.

Since the *C*. *elegans* genome sequence that was available is from a strain not previously investigated for xenobiotic biotransformation (B9769), PCR experiments were conducted using gDNA and cDNA isolated from *C*. *elegans* DSM 1908, which is an established xenobiotic-metabolising strain. The sequences of amplicons obtained matched those of the B9769 strain, confirming the presence of the same genes in 1908. End point RT-PCR experiments demonstrated the expression of eight putative CYPs when the fungus was cultivated in the rich medium sabouraud dextrose broth, which is routinely used in biotransformation experiments. RT-qPCR confirmed that CYP5313D1, identified as coding for a likely xenobiotic-transforming enzyme, was dramatically up-regulated when the fungus was grown in sabouraud dextrose broth compared to RPMI. When *C*. *elegans* was grown in the latter medium the biotransformation of the drug flurbiprofen was very poor in comparison to cells grown in SDB, which was further evidence that CYP5313D1 is an enzyme involved in xenobiotic metabolism in the fungus. Other researchers^15–17^ previously investigated CYP expression in *Cunninghamella* spp. by RT-PCR, using primers based on the conserved haem-binding region of cytochromes P450. Whilst these studies have demonstrated up-regulation of cytochrome P450, they did not take account of the different CYP genes.

Confirmation that CYP5313D1 was capable of hydroxylating a xenobiotic was by heterologous expression of the *cyp5313D1* gene in *P*. *pastoris* and biotransformation of flurbiprofen to the known phase I metabolite 4′-hydroxyflurbiprofen by the recombinant yeast. At present the recombinant system is unoptimised, relying on the endogenous Cpr of *P*. *pastoris* to supply reducing power to the CYP rather than co-expressing a *C*. *elegans* reductase. Future studies will focus on improving product yield through optimising the expression system which will enable a more comprehensive analysis of the enzyme’s properties to be conducted. This is the first xenobiotic-biotransforming cytochrome P450 to be identified in this biotechnologically important fungus and the *in silico* analysis of the Cypome provides a platform for further studies on the other genes.

## Materials and Methods

### Fungal strain and reagents

*Cunninghamella elegans* DSM1908 was obtained from Deutsche Sammlung von Mikroorganismen und Zellkulturen GmbH (DSMZ, Germany) and maintained on sabouraud dextrose agar. Roswell Park Memorial Institute 1640 (RPMI) medium and flurbiprofen were acquired from Sigma-Aldrich, Arklow, Ireland. *Pichia pastoris* X-33 was employed as a fungal host for recombinant protein expression (Fisher Scientific, Dublin, Ireland) and pPICZ A was the vector. YPD medium (Yeast extract Peptone Dextrose) that was used routinely for maintenance and propagation of *P*. *pastoris* X-33 and BMY(G) medium used for protein expression were prepared according to the EasySelect™ Pichia Expression Kit manual. For the preparation of transformed *P*. *pastoris* cells, sorbitol (182.2 g/l) was added to the YPD agar to stabilise the cells to osmotic pressure. Restriction enzymes (New England Biolabs) were purchased from Brennan and Company, Dublin, Ireland. Gibco®Zeocin™ Selection Reagent (100 mg/ml) and T4 DNA Ligase 1 U/µL (Invitrogen) were acquired from Fischer Scientific.

### CYP discovery

Identification of candidate sequences were verified through a multiple sequence alignment tool, MUSCLE^32^, to search for conserved regions among the cytochromes P450. These conserved motifs included the haem-binding domain (FXXGXRXCXG), the PERF domain (PXRX) and the K-helix region (EXXR)^22,33,34^. The genome browser Artemis^35^ was used to visualise the surrounding sequence features to check for errors generated by the gene prediction program. The deduced amino acid sequences were submitted to David Nelson and named according to the International P450 Nomenclature Committee.

### Culture conditions

*Cunninghamella elegans* DSM1908 was cultivated on sabouraud dextrose agar for 120 h at 28 °C. Inoculum was prepared by homogenising the mycelium in sterile 0.8% NaCl (100 ml). The homogenate (5 ml) was used to inoculate 250 ml Erlenmeyer flasks containing 45 ml of either sabouraud dextrose broth (SDB) or RPMI, and then incubated with rotary agitation (150 rpm) at 28 °C.

### Genomic DNA extraction

*C*. *elegans* cultures were grown for 72 h and the biomass (200 mg) was collected aseptically using sterile tweezers after separation from the supernatant by centrifugation (Eppendorf 5810 centrifuge). The biomass was then flash frozen with liquid nitrogen and ground into a powder using a pestle and mortar as described previously^17,36^. The crushed biomass was resuspended immediately in 300 μl of MicroBead Solution from the DNeasy UltraClean Microbial Kit (Qiagen) and processed using the steps outlined in the instruction manual. An increased yield of genomic DNA (gDNA) was achieved by heating the preps at 65 °C before bead beating.

### Total RNA extraction

RNA was extracted from cultures that had been grown in either SDB or RPMI media for 72 h and 96 h. The biomass was collected and handled as described for gDNA isolation. The crushed biomass was resuspended in 1 ml of TRIzol reagent (Invitrogen), vortexed and incubated at room temperature for 5 min. The manufacturer’s instructions were followed and the final pellet was air-dried for 5 min before suspending in diethylpyrocarbonate (DEPC)-treated water (90 μl) and incubated at 65 °C for 5 min. To digest DNA, Turbo DNA-free ^TM^ kit (Invitrogen) was employed, before finally extracting the RNA from the supernatant using an RNeasy mini extraction kit (Qiagen) following the manufacturer’s instructions. Aliquots of the RNA samples were prepared for measuring the final RNA concentration was measured using an Epoch Microplate Spectrophotometer (Biotek) and a Take3 plate (Biotek). RNA samples were stored at −80 °C.

### Preparation of complementary DNA

Template using extracted RNA (100 ng) was prepared on ice and diluted in DEPC treated water to a final volume of 6.1 μl in sterile pre-chilled PCR tubes and random hexamer primers (5 μl) were added. Tubes were incubated at 70 °C for 5 min and then held 4 °C. Reverse transcription was then carried out using an ImProm-II™ reverse transcription system (Promega). Amplification was conducted with a thermocycler (Techne TC-3000 thermocycler) with annealing at 25 °C for 5 min, extension at 42 °C for 1 h followed by heat inactivation at 70 °C for 15 min and then held at 4 °C. Complementary DNA (cDNA) samples were stored at −20 °C for future polymerase chain reactions.

### PCR

Primer design was based on the genome sequence of *C*. *elegans* B9769 for the gDNA and cDNA produced from *C*. *elegans* DSM1908. Oligonucleotide primers were synthesised by Eurofins Genomics (Ebersberg, Germany) and are listed in Table S1. Amplification of target sequences was enabled using either Phusion (New England Biolabs) or *Taq* (Bioline) DNA polymerases. PCR products were routinely purified using a QIAquick PCR Purification Kit (Qiagen) and sent for Sanger DNA sequencing (SUPREMERUN, GATC sequencing). DNA concentrations were measured using the Epoch Microplate Spectrophotometer and Take3 plate. The nucleotide sequence was then aligned with the predicted gene sequence from *C*. *elegans* B9769 using the pairwise sequence alignment tool, EMBOSS Needle. To build full length gene sequences full length and internal overlapping primers were used to ‘primer walk’ to build a curated gene sequence. This was done to search for any incorrect coding sequence data created by the gene prediction (e.g. exon-exon junctions) or any single nucleotide polymorphisms that existed between the bioinformatics strain (*C*. *elegans* B9769) and the experimental strain (*C*. *elegans* DSM1908).

### Quantitative PCR (qPCR)

Quantitative PCR was carried out with the LightCycler 480 SYBR Green I Master using a LightCycler 480 instrument according to the manufacturer’s instructions (Roche LifeScience). Briefly, 15 μl of a PCR mix containing SYBR Green and relevant primers (final concentration 0.5 μM) were aliquoted in a LightCycler 480 multiwell plate-96 and added with 5 ul of cDNA template (previously diluted 1:5). Primers listed in Table S2 were designed to generate amplicons between 100–200 bp. To calculate PCR efficiency, standard curves were prepared using serial dilutions of cDNA (1, 1/4, 1/16 and 1/64). Non-template controls were routinely included in each run. Relevant cycling of parameters is outlined in Table S3. Quantification cycle (Cq) values were obtained using the default settings of the instrument. A final melting curve analysis was performed to demonstrate the formation of a single product. Four experimental conditions were included by growing *C*. *elegans* DSM1908 in SDB or RPMI medium for either 72 h or 96 h. Each experimental condition was represented by at least two biological replicates and each sample was analysed in duplicate. The geNorm method was employed to determine the optimal number of reference targets for normalization^37^. Analysis of data to assess the quantitative variation in gene expression against the control group (RPMI 72), including statistical analysis, was performed in qBaseplus^38^. Log_10_ fold changes (calibrated normalised relative quantities as referred in qBaseplus) were plotted using SigmaPlot 13.

### Heterologous expression of CYP5313D1

The gene encoding CYP5313D1 was amplified from cDNA using primers pαA-8F (5′-ATTCACGTG**CGAAACG**ATGAATGACTTTAATATTTACAATAAATTGGAACAT-3′) and pαA-8R (5′-GAAGTTAAAGCCCAGAAAATTTCCGCGGAATA-3′). Plasmids were digested with *PmlI* and *SacII* prior to ligation to the amplicon. *E*. *coli* DH5α was transformed and positive transformants were selected based on Zeocin™ resistance. Plasmid DNA was purified and the constructs verified by sequencing. Plasmids were purified using a QIAprep Spin Miniprep Kit (Qiagen) and linearised by digesting with *SacI*. The digested DNA was extracted using a phenol:chloroform:isoamyl alcohol (25:24:1) solution and then concentrated by ethanol precipitation (EasySelect™ Pichia Expression Kit manual). Transformation of electro-competent *P*. *pastoris* cells was performed by electroporation (Eppendorf 2510) and transformants were selected by inoculating YPD agar plates containing increasing concentrations of Zeocin™. For protein expression in *P*. *pastoris*, 20–40 ml of BMYG in a baffled 250 ml Erlenmeyer flask was inoculated with a single colony and grown for 48 h at 30 °C, 290 rpm. The culture was then harvested by centrifugation at 4000 rpm for 10 min and the cells washed with 20 ml of BMY medium. The suspension was then centrifuged at 4000 rpm for 10 min and the pellet was resuspended in 40 ml of BMY medium and decanted into a sterile, baffled 250 ml Erlenmeyer flask and incubated for 4 h at 30 °C, 290 rpm. Filter sterilised methanol (0.5% (v/v)) was then added to induce gene expression, with further additions every 24 h. Confirmation of gene expression was by Western blotting. *P*. *pastoris* cells were harvested, resuspended in lysis buffer (50 mM Tris pH 8, 1 mM EDTA, 100 mM NaCl and 1 mM phenylmethylsulfonyl fluoride) and homogenised using BeadBug™. The homogenate was sonicated for 1 min and the lysate finally separated by centrifugation. The soluble and insoluble proteins were separated by SDS-PAGE and the recombinant protein immunodetected using mouse IgG anti-His and anti-myc (BioLegend) and HRP-linked anti-mouse IgG (Cell Signalling Technologies) antibodies; detection was by enhanced chemiluminescence.

### Biotransformation of flurbiprofen

*Cunninghamella elegans* DSM1908 was cultivated in either sabouraud dextrose broth or RPMI for 72 h with rotary agitation (150 rpm) at 28 °C. Flurbiprofen was dissolved in DMF and added to the flasks, which were incubated for a further 72 h. Organic extracts from shake flasks were analysed by reversed-phase high-performance liquid chromatography (HPLC) with a Varian Prostar HPLC system equipped with Zorbax SB-C18 5 μm 4.6 × 150 mm column (Agilent technologies) and a UV–Vis detector monitoring at 250 nm. Samples were injected (25 µl) onto the column and analytes were eluted with a gradient of acetonitrile / water (20–80% acetonitrile) over 35 min at a flow rate of 1 ml/min. Quantification of 4′-hydroxyflurbiprofen was performed by comparing peak areas with a standard curve prepared using previously isolated metabolite^23^.

Whole cell biotransformation experiments with recombinant *P*. *pastoris* cultures were conducted. The cells were cultured as in BMY as described previously and flurbiprofen (1 mg) was added 24 h after induction with methanol. The cultures were incubated for another 48 h then extracted with ethyl acetate; the solvent was removed under reduced pressure and the residue derivatised with *N*-methyl-*N*-(trimethylsilyl)trifluoroacetamide (100 °C for 1 h). The silylated extracts were analysed by gas chromatography-mass spectrometry (GC-MS) as described previously^26^.

## Supplementary information

Supplementary Information

## Data Availability

The datasets generated during and/or analysed during the current study are available from the corresponding author on reasonable request.
