# Supplementary material for: The CYPome of the model xenobiotic-biotransforming fungus Cunninghamella elegans
Source: Sci Rep. 2019 Jun 25;9:9240. doi: 10.1038/s41598-019-45706-x (PMC6592952; doi:10.1038/s41598-019-45706-x)

The CYPome of the model xenobiotic-biotransforming fungus *Cunninghamella elegans*

William Palmer-Brown,[1] Raúl Miranda-CasoLuengo,[1] Kenneth H. Wolfe,[2] Kevin P. Byrne[2] and Cormac D. Murphy[1]*

1. UCD School of Biomolecular and Biomedical Science, University College Dublin, Belfield, Dublin 4, Ireland

2. UCD School of Medicine, Conway Institute, University College Dublin, Belfield, Dublin 4, Ireland

Supplementary Information

Table S1. Primers used to amplify CYP genes from *C. elegans* gDNA and cDNA.

Primers	Nucleotide sequence 5' to 3'	Tm (°C)	CYP
g6049 F	TGGCACGAACAACTTGGTAA	53.2	5206U1
g6049 R	CCAGCAGCAACTAAATCAGC	55.3	
g5706 F	GCTGATTTAGTTGCTGCTGGT	57.9	5208A2
g5706 R	CTAAACCCCGACCAATGGTA	55.9	
g5705 F	GCTGATTTAGTTGCTGCTGGT	55.3	5206D1
g5705 R	GGGAAAAACTCTGCCATTTG	55.3	
g4598 F	TGGCAGAGTTTTTCCCTTTT	55.3	5205A8
g4598 R	TGAATCCCAACACAAACTCG	57.3	
g6826 F	GACTTGCTGTGCAACTTCAAA	57.3	509Q1
g6826 R	TGGTATCTACACCAGCAGCAA	57.3	
g4063 F	GCTGACTTAATTGCTGCTGGT	53.5	5210C1
g4063 R	TGGTAATATAAGCTGCCATTC	55.3	
g3940 F	TGCACCACGTGCTATTAAGG	57.3	5313E1
g3940 R	ACGGGGATGAATGGTCATAA	57.3	
g2620 F	ATGAATGACTTTAATATTTACAATAAATTGGAAC	63.9	5313D1
g2620 R	TTAAAATTTTCTGGGCTTTAACTTCATA	63.8	

Table S2. Primers used in RT-qPCR.

Primers	Nucleotide sequence 5' to 3'	Tm (°C)	GC-content (%)	Ta (°C)
CYP F*	GGTATGAACTTTAGTTTAGAACAA	55.9	25.0	58
CYP R*	CGGATTTTCAAATCAATTGGTTTAGGT	58.9	33.3	58
CPR F*	ATTACTGACGAATCCCCTGT	55.3	45	56
CPR R*	CGTTCACCAATACGTTTAGC	55.3	45	56
SarA F	AACCTACTCTTCATCCTACCTCTGAA	61.6	42.3	60
SarA R	GCATCAACTAAGAAAACATACCAGA	58.5	34.6	60
UbcB F	ATGAGAGATTTCAAGAGACTCCAAA	58.1	36	60
UbcB R	ACAAATTTAACTGATGGTGGTTTGT	56.4	32	60
q-3940 F	ATTTGTTCCTAAAGGCACTAAGGTT	57.3	50	60
q-3940 R	TGAAATTCATTAAATCATTGGGACT	57.3	50	60
q-2620 F	AATTCTACTGGATTTGCTATGATTG	53.5	30.4	60
q-2620 R	TTCATTGATGACAGCATTAAGATAA	55.3	45	60

CYP and CPR primers were specific for CYP509A1 and a cytochrome P450 reductase, respectively.

Table S3. Conditions for RT-qPCR reactions

Step	Temperature (^oC)	Time
Pre-incubation	95 °C	5 min
	95 °C	15 s
45 cycles	~ °C	20 s
	72 °C	10 s
Melting curve	50 to 95 °C	(temperature transition 0.2 °C /s)
Cooling	40 °C	10 s

Table S4. PCR efficiency of amplified targets*

Target	Slope	Y-intercept	E	Error	R2
cyp5313E1	-3.5196	28.12	1.87	0.72	0.94
cyp5313D1	-3.5354	26.31	1.92	0.27	0.99
cyp509A1	-3.2309	29.58	2.04	0.08	1
ubcB	-3.5794	24.5	1.9	0.2	1
sarA	-3.7986	24.62	1.83	0.34	0.99
cpr	-4.2994	25.45	1.71	0.32	0.99

*PCR efficiencies were measured from calibration curves made of serial dilutions of cDNA 1, 1/4, 1/16 and 1/64 measured in duplicate.

Figure S1. Phylogenetic tree of redox partner proteins from *C. elegans* and known CYP partner proteins used for homology searches. Branch bootstrap values are presented as a percentage.

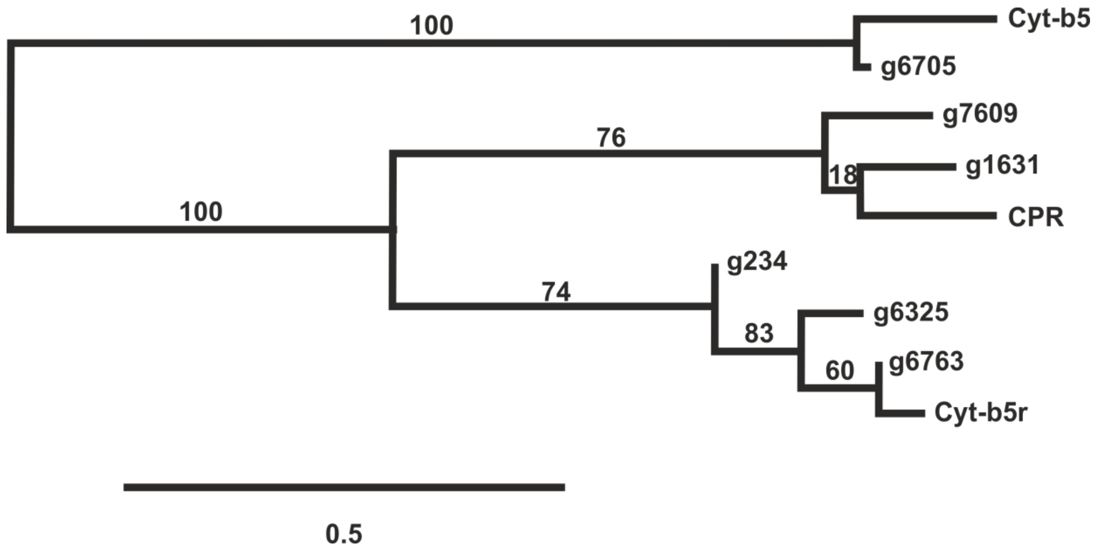

Figure S2. Alignment of the amino acid sequences of CPRs from *C. elegans* (G1631, G7609 and G4301) with those of *Mucor ambiguous* (GAN1069.1) and *Rhizopus stolonifer* (RCH89036.1). FMN domain is in red, NADPH-binding domain is in green and the FAD domain is bold.

```
G1631           --MLKSNNQVFEHQLMLVLGTISVGGLLWVAKGLLFNDSNKSSDQKP--LI--------- 47
G7609           MTTRRSNNRVLGTYHLIVLGAIGLGTIAWFARNKFF----GSSDTDDNKIISNSETSTTS 56
G4301           --MAQQSPAVIDTLDLILLGSIGLGTIAWFTRRQISERLFGTGQSNA------------- 45
GAN10969.1      --MTRSSQHLLDSLDIIFLGTIGLGTIAWFARHQIADKLFKSSKPEI------------- 45
RCH89036.1      --MAKTSHHLLDTLDIVLLGTIGLGTIAWFARHQIANRLFQSNKSSE------------- 45
                    : .  ::    ::.**:*.:* : *.::  :      :.. .

G1631           PDVKTMEKSEEEQEVGNFVKLMKEQNRKVIFFYGSQTGNAENYCYQLSKECKKRYGIQPM 107
G7609           SDTTAPKPNVPAKPERNFVKVMQQQGRRVIFFYGSQTGTAEDYASRLAKECSQKYGISAM 116
G4301           T--SKPTTPQAPKRERNFVKVMEQQGRKVIFFYGSQTGTAEDFASRLAKQCSQKYGVSCM 103
GAN10969.1      KP-ASDAKTGPPKKERNFVKVMQQQGRRVIFFYGSQTGTAEDYASRLAKECSQKYGVSSM 104
RCH89036.1      PNTSTSDEAKSPKKERNFVKVMQEQGRRVIFFYGSQTGTAEDYASRLAKECSQKYGVSAM 105
                            :   ****:*::*.*:**********.**::. :*:*:*.::**:. *

G1631           VADIETYDLKYLDLLTEDNLAVFIVSTYGEGDPTDSAINFWEL-IHNENPNFSKCD-GNP 165
G7609           TADIELYDLNYLDTLPEDNLVFFVMATYGEGEPTDNAVDFWEL-INDETPQFSQLANVED 175
G4301           TADIEMYDLSYLDTLSEDSLVCFVMATYGEGEPTDNAVDFWEQFITDESPVFSQGG---- 159
GAN10969.1      AADIEQYDMSYLDTVPEDFLVFFVMATYGEGEPTDNAVDFWDL-VSEEQPQFSEAE-DEE 162
RCH89036.1      TADIEQYDLSYLDTVPEDCLVFFVVATYGEGEPTDNAVEFWDL-ITDEEPQFSTSD---- 160
                .**** **:.*** : ** *. *:::*****:***.*::**:  : :* * **

G1631           QPLSKLRFFAFGLGNSTYEHFNAAVVGVDRELTRLGATRLGEIGKGDDDACLDDDFAHWQ 225
G7609           KPLKNVRYLVFGLGNKTYEHYNSVGRTVDKKLTDFGATRIGERGEGDDDGSLEEDFLAWQ 235
G4301           ETLENLRYLMFGLGNKTYEHYNAVARILDKKLTGLGAKRIGERGEGDDDGSLEEDFLAWQ 219
GAN10969.1      APLKNLRYVAFGLGNKTYEHYNEVIRNIDKRLTCMGAKRIGERGEGDDDGSLEEDFLAWQ 222
RCH89036.1      -RLENLRYITFGLGNKTYEHYNEVVRKIDHRLLALGAKRIGERGEGDDDGSLEEDFLAWQ 219
                  *.::*:. *****.****:* .   :*:.*  :**.*:** *:****..*::**  **

G1631           ESFWPLFGEAVSQLIDGEADEGNHGDQYAYEVTDVNVDDNDGFYYQGEL---GSDRTQKL 282
G7609           ETMWPAFCQALGIDE---NNIQLGPRQAAFKVEELTTFDDSSVYY-GEIAENLKSKGKIV 291
G4301           ESMWPTFCNALGVDE---NNAQQGPRQASYSVDELEEYQNDDVYF-GELGTISKDSSRVV 275
GAN10969.1      EEMWPAFCEALGVDE---SSASSGPRQASFAVEELTEFDKAKVYV-GELSEWLKEGARVV 278
RCH89036.1      EEMWPAFCEALGVDE---ANAQLGPRQAIFSVEELTEFDKAKVYF-GELSEWLKEGARVV 275
                * :** * :*:.       .      *  : * ::   :.  .*  **:    ..  : :

G1631           FDAKNPYPATVQIRDLTPATQDDRHCLHIDFDLADSGLTYKTGDHLGVWPINNELEVNLV 342
G7609           YDAKRPYNAPIQSRELFQD-GGDRHCLHVDIDISGTNLNYQTGDHVAIWPTNNEIEVIRL 350
G4301           YDAKRPYNAPITTRELFN--SSERHCLHVDIDISGTNLSYQTGDHVAMWPTNNEDEVLRL 333
GAN10969.1      YDAKRPYNAPITSTDLFK--GGDRHCLHMEIDISGSNLTYQTGDHVAIWPTNNEIEIERL 336
RCH89036.1      YDAKRPYNAPITSRDLFK--NSDRHCLHMEIDISDTNLSYQTGDHVAIWPTNSEVEVERL 333
                :***.** * :   :*     .:*****:::*::.:.*.*:****:.:** *.* *:  :

G1631           SSIFGWNHDGILDKVISVKPTDPT--GKVPFPQPTTLRTALRHYLDIAAIPSRSVFELLI 400
G7609           ASILGLKD--KLDTVIMVNAVDSAASKQHPFPVPTTYRTIFRHYLDICSIASRQTLMSLV 408
G4301           ATILGLQE--KLDTVISVKAIDPAAPKQNPFPVPTTYRAIFRHYIDICAPASRQSLMSFV 391
GAN10969.1      AKVLGLSD--KLDTVIRVQALDAAASKQFPFPVPTTYRAIFRHYLDICAAVSRQTLMSLI 394
RCH89036.1      AQVLGLMD--KLDTVIRVQAIDATASKQHPFPVPTTYRAVFRHYLDICAVVPRQTLMSLI 391
                : ::*  .   **.** *:  * :   : *** *** *: :***:**.:   *. :  ::

G1631           PHVP-EQIKAALQKIIDDKEVYNKLVVDEVRNFGQVLSHILTSNGYT---DIENALAKVP 456
G7609           EYAPTEESKANLLRLSKDKDEYHVHVHDSVRNLGEVLQYVCGNNDHTDPEAHSGAFANVP 468
G4301           EFAPTDAAKDLLKLLATDKDEYRLKVGEAVRNLGEVLELVSGKD------AQPGSFSSVP 445
GAN10969.1      EYAPTEKSKDILRKLATDKDEYRVRVGDVTRNLGEVLEMLAESES----LAVEGSFSSVP 450
RCH89036.1      EYAPTEKSKEVLRKLATDKEEYRLRVGDMTRNLGEVLEMLAESES----MDPKNSFANVP 447
                 ..* :  *  *  :  **: *.  * : .**:*:**. :  .:         .:::.**

G1631           INIIIECYTRLQPRYYSISSSSSESGSIVTATAVTLKYNP--TPDRTVYGVNTNYLWSIY 514
G7609           FDLIIESISRLQPRYYSISSSSKEQPNIISATCVTLEYQPKPTPERTVYGVNTNYLWKIH 528
G4301           FDLIVETIPRLQPRYYSISSSSKENPSIISATCVTLAYQPDPTPDRTVYGVNTNFLYRIH 505
GAN10969.1      FDLIVESVSRLQPRYYSISSSSKESPKKITVTVVTLQYTPETSSPRTVYGVNTNYLWRLH 510
RCH89036.1      FDLIVEAVSRLQPRYYSISSSSKEDPKKITVTAVTLQYTPDLSSPRTVYGVNTTYLWRLH 507
                :::*:*   *************.*. ::.* *** * *  :  ********.:*: ::
```

```
G1631           QKLNPSVANPNHPPYAIEGPHQAYFQKNGDTIITKLPVHIRQSTFRLPTDASTPVVMVGP  574
G7609           SIVHQVDDGRSYPHYDIAGPRQQLFESSL--SHAKIPIHVRRSQFKLPRNSKVPVIMVGP  586
G4301           MQNNDDSVIQGLPKYDLAGPRKAFLNGQG--QSHKLPIHIRRSQFKLPRNTSCPVIMIGP  563
GAN10969.1      ESINGIEPDASFPHYSITGPRNCLYDTEA--KAARVPVHVRRSQFKLPRNPTVPIIMVGP  568
RCH89036.1      EAINGLEPDTASPRYYLGGPRDGLFDVQA--KQARVPVHVRRSQFKLPRNPTVPIIMVGP  565
                   :        * * : **:.    : .      ::*:*:*:* *:** : . *::*:**

G1631           GTGVAPFRGFVRERVYQKQVEKQQVGTTLLFFGCRRSNEDYLYADEWPELFNQLDNESRI  634
G7609           GTGVAPFRGFVRERALLKQK-GQEVGPTVLFFGCRHSEKDFIYKDEWPELFNTLGEESQL  645
G4301           GTGVAPFRGFVRERALQKKE-GKSVGPTILFFGNRHSQHDFLYSDEWPELFNTLGEDSKL  622
GAN10969.1      GTGVAPFRGFVRERALQKKE-GKPIGPTILYFGCRNSAEDFLYEEEWPELFETLGESSRI  627
RCH89036.1      GTGVAPFRGFVRERALQKKE-GKPVGTTVLFFGCRHPEQDFLYADEWPELFETLGGDSRL  624
                **************.  *:    : :* *:*:** *.   .*::* :******: *. .*::

G1631           INAFSRETDKKVYVQHRVRENGEEVWDLLANQGGYLYVCGDAKRMAKDITQTILDLAKHF  694
G7609           ITAFSRETDQKVYVQHRLKEYGQQMWDYI-QQGAYIYVCGDAKNMAHDVNQAFIQFAQEF  704
G4301           ITAFSRESEHKVYVQHRLEENGKEIWQLL-EKGAYIYVCGDARNMARDVNQTFVNLAMEY  681
GAN10969.1      ITAFSRETAQKVYVQHRLMENGPEMWDLL-EKGAYVYVCGDAKVMARDVNQTFVRFAQQF  686
RCH89036.1      ITAFSRETDKKVYVQHRLLEHGQAMWNLL-EKGAYVYVCGDAKVMARDVNQTFVQFAKDL  683
                *.*****: :*******: * *  :*: : ::*.*:******: **:*:.*::: :* .

G1631           GKLDDEAALAFVQGLRKAGRYQEDVWA       721
G7609           GGRDEVKANAFFKQLRNTGRYQEDVWS       731
G4301           GEKTEQKALDYVKSLRNTGRYQEDVWS       708
GAN10969.1      GGHDEEKAQDYVKNLRNTGRYQEDVWS       713
RCH89036.1      GGLDEAEAQDYVKNLRNTGRYQEDVWA       710
                *    :  *   :.: **::********:
```

Figure S3. Amplification of *cyp5313D1* from cDNA of *C. elegans* DSM1908. The expected amplicon size was 1,539 bp.

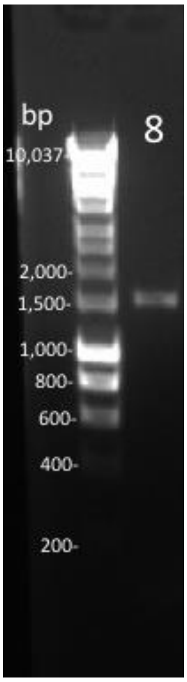

Figure S4. Full Western blot corresponding to Fig 3. The upper blot was developed with anti-his antibodies and the lower blot probed with anti-myc antibodies.

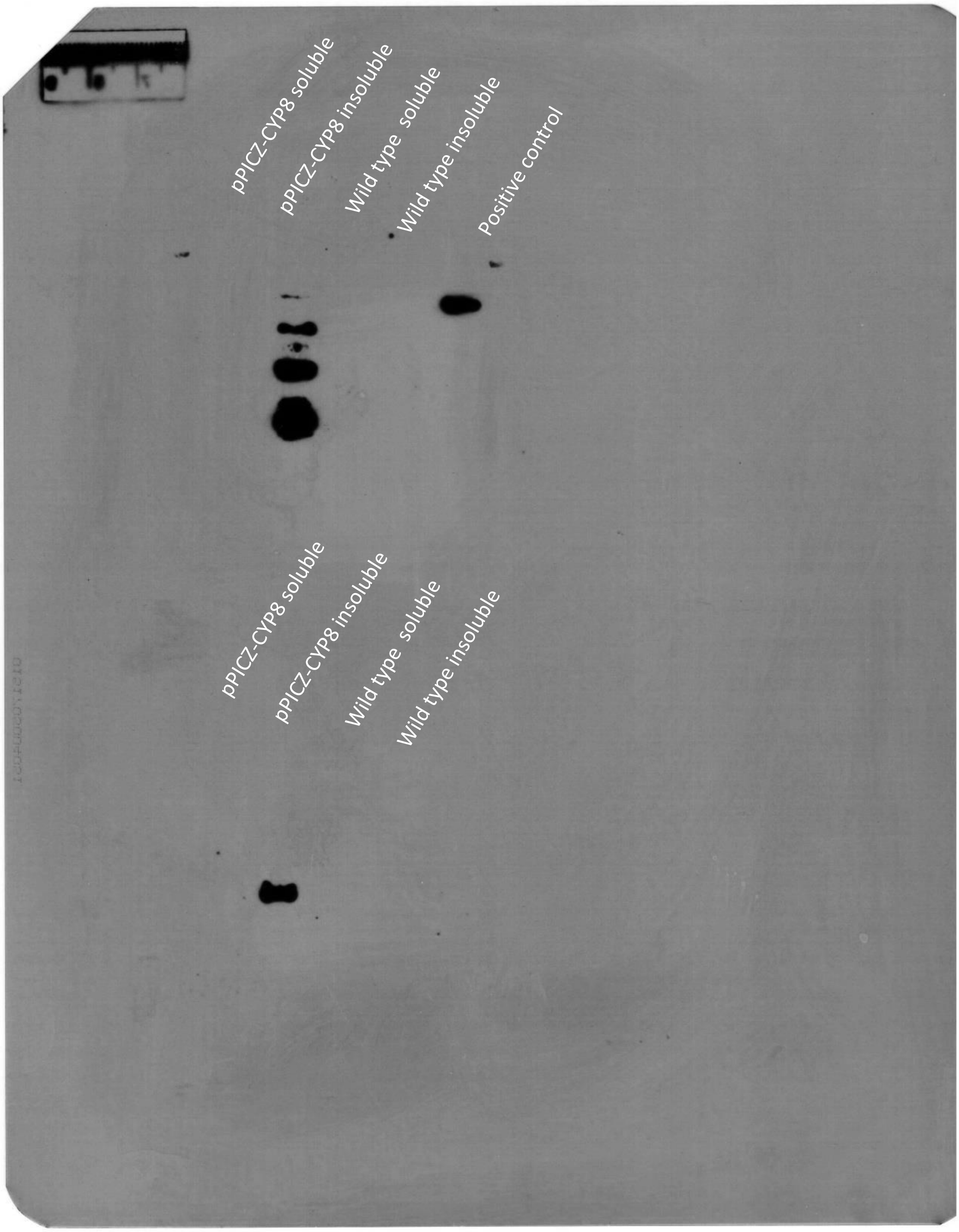

Supplement: Supplementary file 1 — Supplementary Information [file 41598_2019_45706_MOESM1_ESM.pdf]
